# Supplementary material for: Eating Disorder Risk Among Adolescents: The Influence of Dietary Patterns, Physical Activity, and BMI
Source: Nutrients. 2025 Mar 19;17(6):1067. doi: 10.3390/nu17061067 (PMC11946152; doi:10.3390/nu17061067)
Supplement: Supplementary file 1 [file nutrients-17-01067-s001.zip › nutrients-3501196-supplementary.pdf]

Supplementary Table S1: Item-Level Distribution of Responses to the Eating Attitudes Test-26 (EAT-26)

Variable	Response Category	Frequency (n)	Valid Percent (%)
**I am terrified about being overweight**	Sometimes, Rarely, Never	201	47.5
	Often	43	10.2
	Usually	62	14.7
	Always	117	27.7
**I avoid eating when I am hungry**	Sometimes, Rarely, Never	310	73.3
	Often	46	10.9
	Usually	54	12.8
	Always	13	3.1
**I find myself preoccupied with food**	Sometimes, Rarely, Never	239	56.5
	Often	65	15.4
	Usually	68	16.1
	Always	51	12.1
**I have gone on eating binges where I feel that I may not be able to stop**	Sometimes, Rarely, Never	321	75.9
	Often	49	11.6
	Usually	35	8.3
	Always	18	4.3
**I cut my food into small pieces**	Sometimes, Rarely, Never	306	72.3
	Often	54	12.8
	Usually	38	9.0
	Always	25	5.9
**I am aware of the calorie content of the foods that I eat**	Sometimes, Rarely, Never	232	54.8
	Often	52	12.3
	Usually	66	15.6
	Always	73	17.3
**I avoid food with a high carbohydrate content**	Sometimes, Rarely, Never	338	79.9
	Often	35	8.3
	Usually	31	7.3
	Always	19	4.5
**I feel that others would prefer if I ate more**	Sometimes, Rarely, Never	285	67.4
	Often	41	9.7
	Usually	49	11.6
	Always	48	11.3
**I vomit after I have eaten**	Sometimes, Rarely, Never	382	90.3
	Often	15	3.5
	Usually	12	2.8
	Always	14	3.3

**I feel extremely guilty after eating**	Sometimes, Rarely, Never	337	79.7
	Often	24	5.7
	Usually	28	6.6
	Always	34	8.0
**I am preoccupied with a desire to be thinner**	Sometimes, Rarely, Never	276	65.2
	Often	48	11.3
	Usually	36	8.5
	Always	63	14.9
**I think about burning up calories when I exercise**	Sometimes, Rarely, Never	249	58.9
	Often	60	14.2
	Usually	47	11.1
	Always	67	15.8
**Other people say I am too thin**	Sometimes, Rarely, Never	290	68.6
	Often	40	9.5
	Usually	47	11.1
	Always	46	10.9
**I am preoccupied with the thought of having fat on my body**	Sometimes, Rarely, Never	271	64.1
	Often	55	13.0
	Usually	35	8.3
	Always	62	14.7
**I take longer than others to eat my meals**	Sometimes, Rarely, Never	283	66.9
	Often	45	10.6
	Usually	52	12.3
	Always	43	10.2
**I avoid foods with sugar in them**	Sometimes, Rarely, Never	318	75.2
	Often	47	11.1
	Usually	36	8.5
	Always	22	5.2
**I eat diet foods**	Sometimes, Rarely, Never	314	74.2
	Often	55	13.0
	Usually	28	6.6
	Always	26	6.1
**I feel that food controls my life**	Sometimes, Rarely, Never	326	77.1
	Often	36	8.5
	Usually	38	9.0
	Always	23	5.4
**I display self-control around food**	Sometimes, Rarely, Never	216	51.1
	Often	76	18.0
	Usually	68	16.1
	Always	63	14.9

**I feel that others pressure me to eat**	Sometimes, Rarely, Never	309	73.0
	Often	49	11.6
	Usually	37	8.7
	Always	28	6.6
**I give too much time and thought to food**	Sometimes, Rarely, Never	316	74.7
	Often	38	9.0
	Usually	34	8.0
	Always	35	8.3
**I feel uncomfortable after eating sweets**	Sometimes, Rarely, Never	306	72.3
	Often	39	9.2
	Usually	40	9.5
	Always	38	9.0
**I engage in dieting behavior**	Sometimes, Rarely, Never	308	72.8
	Often	46	10.9
	Usually	32	7.6
	Always	37	8.7
**I like my stomach to be empty**	Sometimes, Rarely, Never	323	76.4
	Often	34	8.0
	Usually	29	6.9
	Always	37	8.7
**I have the impulse to vomit after meals**	Sometimes, Rarely, Never	342	80.9
	Often	46	10.9
	Usually	26	6.1
	Always	9	2.1
**I enjoy trying new rich foods**	Always, Usually, Often	190	44.9
	Sometimes	94	22.2
	Rarely	74	17.5
	Never	65	15.4
**Episodes of excessive eating in the past 6 months**	Never	190	44.9
	Once a month or less	110	26.0
	2-3 times a month	63	14.9
	Once a week	33	7.8
	2-6 times a week	17	4.0
	Once a day or more	10	2.4
**I made myself vomit to control my weight or shape in the past 6 months**	Never	346	81.8
	Once a month or less	32	7.6
	2-3 times a month	21	5.0
	Once a week	13	3.1
	2-6 times a week	4	0.9
	Once a day or more	7	1.7

**I used laxatives, diet pills, or diuretics to control my weight or shape in the past 6 months**	Never	360	85.1
	Once a month or less	24	5.7
	2-3 times a month	17	4.0
	Once a week	8	1.9
	2-6 times a week	5	1.2
	Once a day or more	9	2.1
**I exercised more than 60 minutes a day to lose or control my weight in the past 6 months**	Never	136	32.2
	Once a month or less	55	13.0
	2-3 times a month	57	13.5
	Once a week	42	9.9
	2-6 times a week	82	19.4
	Once a day or more	51	12.1
**I lost 9 kg or more in the past 6 months**	No	414	97.9
	Yes	9	2.1

Note. Each item's total frequency is 423 (100.0%).

Supplementary Table S2: Item-Level Distribution of Responses to the Medi-Lite score

Variable	**Category**	**Frequency (n)**	**Percent (%)**
Vegetable Consumption (portions/day)	<1	153	36.2
	1-2.5	163	38.5
	>2.5	107	25.3
Legume Consumption (portions/week)	<1	241	57
	1-2	105	24.8
	>2	77	18.2
Fruit Consumption (portions/day)	<1	170	40.2
	1-2	156	36.9
	>2	97	22.9
Cereal Consumption (portions/day)	<1	182	43
	1-1.5	125	29.6
	>1.5	116	27.4
Dairy Consumption (portions/day)	<1	178	42.1
	1-1.5	155	36.6
	>1.5	90	21.3
Fish Consumption (portions/week)	<1	206	48.7
	1-2.5	103	24.3
	>2.5	114	27
Meat Consumption (portions/day)	<1	84	19.9
	1-1.5	127	30
	>1.5	212	50.1
Olive Oil Use	Occasional	173	40.9
	Frequent	134	31.7
	Regular	116	27.4
